# Real-world safety and effectiveness of nivolumab for recurrent or metastatic head and neck cancer in Japan: a post-marketing surveillance

**DOI:** 10.1007/s10147-021-01949-1

**Published:** 2021-06-10

**Authors:** Makoto Tahara, Naomi Kiyota, Ken-ichi Nibu, Ayumi Akamatsu, Tomohiro Hoshino, Ryuichi Hayashi

**Affiliations:** 1 Department of Head and Neck Medical Oncology, National Cancer Center Hospital East, 6-5-1 Kashiwanoha, Kashiwa, Chiba 277-8577 Japan; 2 Department of Medical Oncology and Hematology, Kobe University Hospital, 7-5-2 Kusunoki-cho, Chuo-ku, Kobe, Hyogo 650-0017 Japan; 3 Cancer Center, Kobe University Hospital, 7-5-2 Kusunoki-cho, Chuo-ku, Kobe, Hyogo 650-0017 Japan; 4 Department of Otolaryngology-Head and Neck Surgery, Kobe University School of Medicine, 7-5-2 Kusunoki-cho, Chuo-ku, Kobe, Hyogo 650-0017 Japan; 5 Post Marketing Surveillance Pharmacovigilance Department, Ono Pharmaceutical Co., Ltd., 2-1-5 Dosho-machi, Chuo-ku, Osaka, 541-8526 Japan; 6 Safety Management Pharmacovigilance Department, Ono Pharmaceutical Co., Ltd., 2-1-5 Dosho-machi, Chuo-ku, Osaka, 541-8526 Japan; 7 Department of Head and Neck Surgery, National Cancer Center Hospital East, 6-5-1 Kashiwanoha, Kashiwa, Chiba 277-8577 Japan

**Keywords:** Nivolumab, Head and neck cancer, Post-marketing surveillance

## Abstract

**Background:**

On the basis of phase III CheckMate 141 results, nivolumab was approved for recurrent or metastatic head and neck cancer after undergoing platinum-containing chemotherapy in Japan. This post-marketing surveillance aimed to evaluate the safety and effectiveness of nivolumab for head and neck cancer in the real-world setting.

**Methods:**

All patients with head and neck cancer who planned to receive nivolumab were centrally registered. This study monitored 607 patients for 6 months to assess nivolumab’s safety, especially treatment-related adverse events (TRAEs) of special interest, and effectiveness.

**Results:**

TRAEs occurred in 36.1% patients, with no new safety signals. The most common TRAEs with grade ≥ 3 were interstitial lung disease (1.2%), diarrhea (0.8%), and hepatic function abnormal (0.7%). Meanwhile, thyroid dysfunction (10.2%), hepatic dysfunction (5.3%), and interstitial lung disease (4.1%) were the most common TRAE categories of special interest. Although the median time to the onset of each TRAE category of special interest was mostly 1–2 months, most of them occurred throughout the observation period; nonetheless, the majority of patients recovered or remitted. The 6-month survival rate was 55.9%.

**Conclusion:**

Japanese patients with head and neck cancer treated with nivolumab in the real-world setting manifested no new safety signals.

**Clinical Trial Registration:**

clinicaltrials.jp: JapicCTI-184071.

**Supplementary Information:**

The online version contains supplementary material available at 10.1007/s10147-021-01949-1.

## Introduction

Nivolumab is the first humanized monoclonal antibody against human programmed death-1 (PD-1) [1]. Nivolumab is an immune checkpoint inhibitor with anticancer activities; it restores the immune function of cytotoxic T-lymphocytes, thereby eliminating tumor cells [2, 3]. In July 2014, nivolumab was approved for melanoma in Japan, and subsequently for treatment of diverse cancer types in more than 60 countries.

CheckMate 141, which is a randomized phase III study, evaluated nivolumab in patients with recurrent or metastatic squamous cell carcinoma of the head and neck that had been progressed or recurred within 6 months after undergoing platinum-containing chemotherapy. In CheckMate 141, nivolumab significantly improved patients’ overall survival compared with a standard chemotherapy drug of investigator’s choice including cetuximab, docetaxel, and methotrexate [4, 5]. On the basis of these results, nivolumab was approved for treating patients with recurrent or metastatic head and neck cancer after undergoing platinum-containing chemotherapy in Japan. However, only 18 Japanese patients were enrolled for nivolumab treatment in CheckMate 141; therefore, extensive evaluation of nivolumab’s efficacy and safety is necessary for Japanese patients with head and neck cancer. As part of the approval condition, the Japanese Ministry of Health, Labor and Welfare requested the manufacturer to conduct a post-marketing surveillance for all cases of nivolumab treatment for head and neck cancer. Thus, this prospective study evaluated the safety and effectiveness of nivolumab treatment in patients with head and neck cancer in the real-world setting in Japan. In particular, we evaluated the incidence of treatment-related adverse events (TRAEs) of special interest that are specified in the Risk Management Plan of nivolumab based on previous clinical trials and post-marketing surveillance [4, 6]. We also explored potential risk factors for TRAEs of special interest. Recently, a few retrospective studies with 100–250 patients reported that nivolumab is effective and safe for head and neck cancer [7–9]. Here, we report the safety and effectiveness of nivolumab in more than 600 Japanese patients with head and neck cancer.

## Patients and methods

### Study design and patients

This prospective, non-interventional, observational study evaluated the safety and effectiveness of nivolumab treatment for 6 months after the first dose in Japanese patients with head and neck cancer. This study conformed to the Japanese Good Post-Marketing Study Practice regulations. Each participating hospital agreed to contracts for this surveillance with the study sponsor. No intervention was made for the purpose of this study; therefore, no written informed consent was required from each patient. This study was registered at clinicaltrials.jp: JapicCTI-184071.

In line with the nivolumab label in Japan, patients with recurrent or metastatic head and neck cancer who had previously received platinum-containing chemotherapy are eligible for nivolumab treatment. All patients with head and neck cancer who planned to receive nivolumab treatment were centrally registered from the approval date of March 24, 2017. Although the registration is planned to continue until March 2021, we analyzed case report forms collected from patients who received nivolumab by June 30, 2017; on this date, the planned number of patients was already reached. In this report, we assessed case report forms from 224 hospitals that permitted data publication.

Patients intravenously received a nivolumab dose of 3 mg/kg every 2 weeks. Then, they were monitored for 6 months after the first dose. Patients who discontinued nivolumab treatment within 6 months were also followed up, if possible, for 6 months after the first nivolumab dose.

### Assessments

We collected patients’ baseline demographic characteristics, including sex, age, Eastern Cooperative Oncology Group performance status (ECOG PS), smoking and alcohol history, medical history, cancer stage, primary tumor site, and previous treatments. Nivolumab administration conditions such as the duration of administration, the number of dose, and the reason for discontinuation were also collected.

The primary outcome measurement was the incidence of TRAEs. We used the Japanese version of the Medical Dictionary for Regulatory Activities version 22.1 and the National Cancer Institute Common Terminology Criteria for Adverse Events version 4.0 for classifying and grading each TRAE, respectively. Furthermore, we evaluated the incidences of TRAEs in patient subpopulations stratified by baseline characteristics. The Risk Management Plan of nivolumab enumerated the following as the TRAE categories of special interest: interstitial lung disease (ILD); myasthenia gravis, myocarditis, myositis, and rhabdomyolysis; colitis and severe diarrhea; type 1 diabetes mellitus; hepatic dysfunction; thyroid dysfunction; neurological disorder; renal disorder; adrenal disorder; encephalitis; severe skin disorder; venous thromboembolism; infusion reaction within 24 h after administration; immune thrombocytopenic purpura; and cardiac disorder such as atrial fibrillation, bradycardia, or ventricular extrasystole.

In addition, the overall survival (OS) rate at 6 months was calculated as the proportion of patients confirmed to be alive 6 months after the first nivolumab dose.

### Statistical analysis

The planned number of patients was 400, which was required to evaluate the incidence of ILD. ILD such as pneumonitis is the most critical TRAE that may result in fatality; thus, it has been cautioned in the nivolumab label. Considering 2.12% of patients in CheckMate 141 had pneumonitis [4], an analysis with 400 patients could detect the same incidence with 95% confidence interval (CI) of 0.95–4.06%. Moreover, given that the least incidence of TRAEs of special interest was 0.42% in CheckMate 141 and the statistical power to detect the TRAE within 400 patients is 81.43%, TRAEs of special interest other than ILD may be evaluable with the planned number of patients.

The 95% CIs for TRAE incidences and 6-month OS rate were calculated by Fisher’s exact test. The incidences of TRAEs in patient subgroups were compared appropriately using the Fisher’s exact test, the Wilcoxon rank sum test, or the Chi-squared test. Moreover, risk factors for ILD, hepatic dysfunction, and thyroid dysfunction were evaluated among 34 variables of patients’ baseline characteristics by univariate and multivariate analyses using the Fine-Gray proportional subdistribution hazards model, and any decease before the TRAE onset was a competitive risk. The hazard ratio (HR) and 95% CI were estimated using the Fine-Gray subdistribution hazards model with determined risk factors. SAS statistical software (SAS Institute Japan Ltd.) version 9.4 TS1M4 was used for all statistical analyses.

## Results

### Patients

**Fig. 1:**
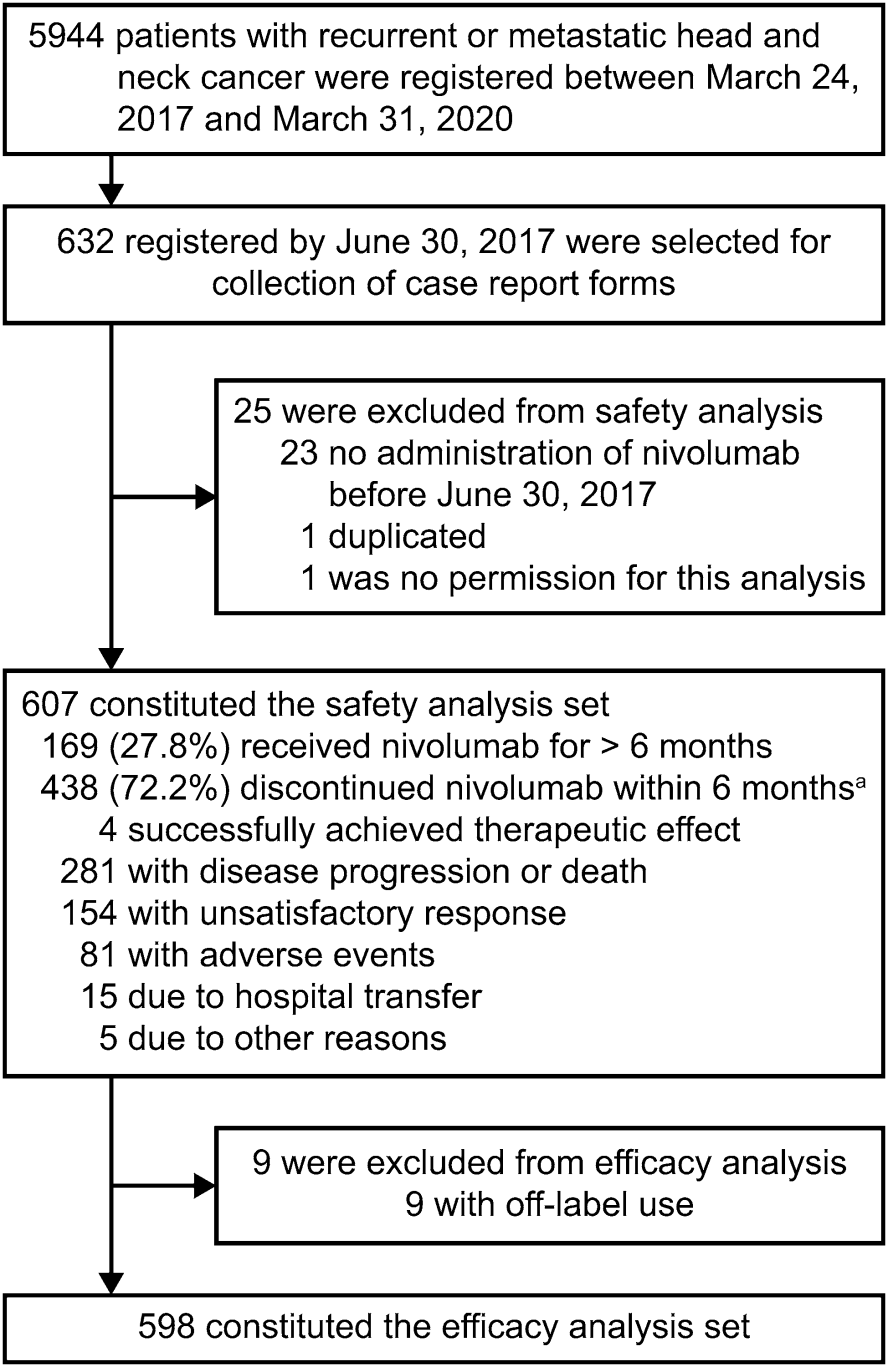
Patient disposition. ^a^Multiple reasons for nivolumab discontinuation could be provided

From March 24, 2017, to March 31, 2020, 5944 patients with head and neck cancer were registered; we analyzed 632 patients who planned to start nivolumab treatment by June 30, 2017, because the prespecified number of patients to be included in the study were assembled by then (Fig. 1). Of those, 24 were excluded from the safety analysis, predominantly because they did not receive nivolumab by June 30, 2017, and one was excluded because of the lack of permission for this report. Thus, the case report forms of 607 eligible patients were analyzed as the safety analysis set. In this set, nine patients were excluded from the effectiveness analysis, because they had not received platinum-containing chemotherapy before the nivolumab treatment; ultimately, 598 patients constituted the effectiveness analysis set.

In the safety analysis set, 454 patients (74.8%) were male (Table 1). The median age was 64 years, and 76 (12.5%) were ≥ 75 years. Most patients (78.7%) had ECOG PS 0 or 1; meanwhile, 129 patients (21.3%) had ECOG PS ≥ 2, which is an ineligibility criterion for CheckMate 141, and 40 patients (6.6%) had ECOG PS ≥ 3. The majority (84.5%) suffered from stage IV head and neck cancer, with the pharynx as the most common primary site (47.0%).

**Table 1:** Baseline patient characteristics and treatment with nivolumab (*N* = 607)

Characteristics	Patients
Sex—*n* (%)	
Male	454 (74.8)
Female	153 (25.2)
Age—years	
Median	64
Range	23–91
Age groups—*n* (%)	
≤ 64 years	315 (51.9)
65–74 years	216 (35.6)
≥ 75 years	76 (12.5)
ECOG PS—*n* (%)	
0	190 (31.3)
1	288 (47.4)
2	89 (14.7)
3	36 (5.9)
4	4 (0.7)
Smoking history—*n* (%)	
Yes	408 (67.2)
No	156 (25.7)
Unknown	43 (7.1)
Alcohol history—*n* (%)	
Yes	381 (62.8)
No	159 (26.2)
Unknown	67 (11.0)
Medical history—*n* (%)	
Yes	363 (59.8)
Lung	105 (17.3)
ILD	9 (1.5)
Emphysema/COPD	15 (2.5)
Lung infection	19 (3.1)
Liver	50 (8.2)
Kidney	32 (5.3)
Thyroid	74 (12.2)
Autoimmune disease	13 (2.1)
No	242 (39.9)
Unknown	2 (0.3)
Cancer stage—*n* (%)	
III	22 (3.6)
IV	513 (84.5)
Recurrence	45 (7.4)
Unknown	27 (4.4)
Tumor site^a^—*n* (%)	
Oral cavity	149 (24.5)
Maxillary sinus	32 (5.3)
Nasopharynx	43 (7.1)
Oropharynx	96 (15.8)
Hypopharynx	146 (24.1)
Larynx	46 (7.6)
Others	168 (27.7)
Treatment lines—*n* (%)	
1st	2 (0.3)
2nd	126 (20.8)
≥ 3rd	475 (78.3)
Unknown	4 (0.7)
Treatment history—*n* (%)	
Cetuximab	390 (64.3)
Paclitaxel	217 (35.7)

*COPD* chronic obstructive pulmonary disease; *ECOG PS* Eastern Cooperative Oncology Group performance status; *ILD* interstitial lung disease

^a^Patients may be overlapped among categories

### Treatments

The median frequency of nivolumab administration was 6 (range 1–18). The average dose of each administration was 157.7 (SD 32.6) mg and 3.0 (SD 0.03) mg/kg. The median duration of administration was 85 (range 1–302) days.

In the safety analysis set, nivolumab treatment was continued in 169 patients (27.8%) for ≥ 6 months but discontinued in 438 patients (72.2%) within 6 months (Fig. 1). Major reasons for discontinuation were as follows: a successful nivolumab response in 4 patients, disease progression or death in 281, unsatisfactory response in 154, and adverse events in 81.

### Safety

The incidence of any TRAEs was 36.1%, and that of grade ≥ 3 TRAEs was 12.5% (Table 2). The most common TRAE was hypothyroidism (7.6%) (Online Resource 1: Supplementary Table S1), while the most common grade ≥ 3 TRAEs were ILD (1.2%), diarrhea (0.8%), and hepatic function abnormal (0.7%). Possible relationships between death and the nivolumab treatment could not be ruled out in 16 patients (2.6%) (Table 2).

**Table 2:** Treatment-related adverse events

Treatment-related adverse events	Number (%) of patients (*N* = 607)		
	Any grades	Grade 3 or 4	Grade 5^g^
Any^a^	219 (36.1)	60 (9.9)	16 (2.6)
Endocrine disorder	62 (10.2)^b^	7 (1.2)	0
General disorders and administration site conditions	39 (6.4)^c^	1 (0.2)	2 (0.3)
Respiratory, thoracic and mediastinal disorders	37 (6.1)	12 (2.0)	6 (1.0)^h^
Skin and subcutaneous tissue disorders	37 (6.1)^d^	6 (1.0)	0
Investigations	36 (5.9)^e^	8 (1.3)	0
Gastrointestinal disorders	34 (5.6)^f^	11 (1.8)	3 (0.5)
Metabolism and nutrition disorders	19 (3.1)^e^	8 (1.3)	0
Hepatobiliary disorders	16 (2.6)^e^	7 (1.2)	2 (0.3)
Nervous system disorders	13 (2.1)^e^	2 (0.3)	0
Musculoskeletal and connective tissue disorders	11 (1.8)	3 (0.5)	0
Infections and infestations	9 (1.5)^e^	2 (0.3)	1 (0.2)^h^
Injury, poisoning and procedural complications	8 (1.3)	1 (0.2)	0
Renal and urinary disorders	6 (1.0)	2 (0.3)	0
Blood and lymphatic system disorders	5 (0.8)	3 (0.5)	0
Cardiac disorders	5 (0.8)	0	2 (0.3)
Neoplasms benign, malignant and unspecified	4 (0.7)	1 (0.2)	1 (0.2)
Psychiatric disorders	2 (0.3)	1 (0.2)	0
Eye disorders	2 (0.3)	0	0
Ear and labyrinth disorders	1 (0.2)	0	0
Reproductive system and breast disorders	1 (0.2)	0	0

^a^The number of patients with any treatment-related adverse events is shown

^b^The grades of two events were unknown

^c^The grades of four events were unknown

^d^The grades of five events were unknown

^e^The grade of one event was unknown

^f^The grades of three events were unknown

^g^Grade 5 treatment-related adverse events included death, general physical condition worsened, asphyxia, interstitial lung disease (2 patients), pleural effusion, pneumonia aspiration, respiratory failure, hematemesis (2 patients), upper gastrointestinal hemorrhage, cholangitis sclerosing, hepatic hemorrhage, sepsis, cardiac arrest, cardio-respiratory arrest hemorrhagic, and tumor necrosis

^h^One patient died because of interstitial lung disease and sepsis

Online Resource 1: Supplementary Table S2 summarizes the incidences of TRAEs in patient subpopulations classified by patients’ baseline characteristics. Patients with medical history had a higher TRAE incidence than those without medical history (42.7% vs. 26.4%). Regarding medical history for each organ, patients with medical history of hepatic, renal, or thyroidal diseases had higher TRAE incidences than those without the corresponding medical history. With regard to lung medical history, the incidence of TRAEs demonstrated no statistically significant difference. Moreover, patients with hepatic medical history had a higher incidence of hepatobiliary disorders including hepatic function abnormal than those without hepatic medical history (6.0% vs. 2.3%) (Online Resource 1: Supplementary Table S3). Likewise, the incidence of endocrine disorders including hypothyroidism and thyroid disorder was higher in patients with thyroidal medical history (20.3%) than those without thyroidal medical history (8.9%). For renal medical history, hypothyroidism was the only TRAE observed in three or more patients (9.4%).

**Fig. 2:**
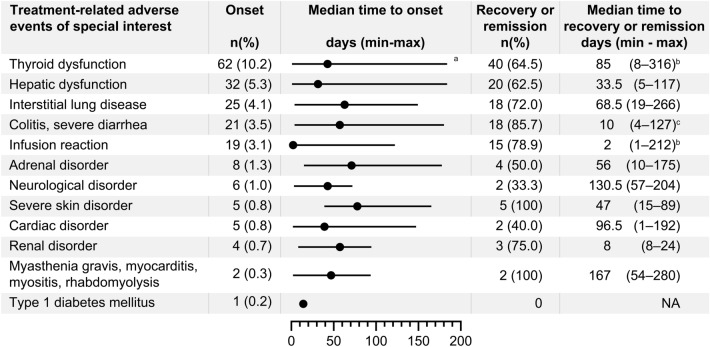
Treatment-related adverse events of special interest. Incidence, median time to onset, the number of patients who recovered or remitted, and median time to recovery or remission are shown. ^a^The timing of onset was unknown in one patient. ^b^The timing of recovery or remission was unknown in one patient. ^c^The timing of recovery or remission was unknown in two patients. *NA* not applicable

The most common TRAEs of special interest were thyroid dysfunction in 62 (10.2%), hepatic dysfunction in 32 (5.3%), and ILD in 25 (4.1%) patients (Table 3). The incidence of these TRAE categories was ≥ 1 percentage point higher than those reported in the whole population of CheckMate 141 (Online Resource 1: Supplementary Table S4). No incidences of encephalitis, venous thromboembolism, or immune thrombocytopenic purpura were reported. Online Resource 1: Supplementary Table S5 lists the exploratory analysis-identified risk factors for these common TRAE categories. Current or former smoking history was a possible risk factor for thyroid dysfunction (HR 2.38) and hepatic dysfunction (HR 3.10). Patients with a medical history of pulmonary infection (HR 5.11) or with a medical history of emphysema or chronic obstructive pulmonary disease (HR 5.64) had a high risk of developing ILD. Although the median time to the onset of each TRAE of special interest were mostly 1–2 months, most of the TRAEs occurred throughout the observation period of 6 months (Fig. 2). Nonetheless, with appropriate treatment including steroid treatment, most patients recovered or remitted from TRAEs of special interest (Table 4).

**Table 3:** Treatment-related adverse events of special interest

Categories	Number (%) of patients (*N* = 607)		
	Any grades	Grade 3 or 4	Grade 5
Thyroid dysfunction	62 (10.2)^a^	4 (0.7)	0
Hepatic dysfunction	32 (5.3)^b^	11 (1.8)	0
Interstitial lung disease	25 (4.1)	7 (1.2)	2 (0.3)
Colitis, severe diarrhea	21 (3.5)^a^	7 (1.2)	0
Infusion reaction	19 (3.1)^a^	0	1 (0.2)
Adrenal disorder	8 (1.3)	2 (0.3)	0
Neurological disorder	6 (1.0)	0	0
Severe skin disorder	5 (0.8)	5 (0.8)	0
Cardiac disorder	5 (0.8)	0	2 (0.3)
Renal disorder	4 (0.7)	1 (0.2)	0
Myasthenia gravis, myocarditis, myositis, rhabdomyolysis	2 (0.3)	1 (0.2)	0
Type 1 diabetes mellitus	1 (0.2)	1 (0.2)	0

^a^The grades of two events were unknown

^b^The grade of one event was unknown

**Table 4:** Treatments and outcomes of treatment-related adverse events of special interest

Categories	Outcomes			
	Recovered or remitted	Not recovered	Died	Unknown
Thyroid dysfunction^a^ (*N* = 62)	42 (67.7)^b^^,c^	18 (29.0)	0	2 (3.2)
Without any treatments	12	4	0	2
Treated with HRT	27^c^	12	0	0
Treated with HRT and corticosteroids	1	0	0	0
Treated with others	1	1	0	0
Hepatic dysfunction^d^ (*N* = 32)	20 (62.5)	11 (34.4)	0	1 (3.1)
Without any treatments	14	8	0	1
Treated with corticosteroids	4	2	0	0
Treated with others	2	0	0	0
Interstitial lung disease (*N* = 25)	18 (72.0)	3 (12.0)	2 (8.0)	2 (8.0)
Without any treatments	3	1	0	0
Treated with corticosteroids	11	1	2	2
Treated with others	4	1	0	0
Colitis, severe diarrhea^a^ (*N* = 21)	18 (85.7)	2 (9.5)	0	1 (4.8)
Without any treatments	5	0	0	0
Treated with corticosteroids	6	0	0	0
Treated with others	5	2	0	1
Infusion reaction (*N* = 19)	15 (78.9)	3 (15.8)	1 (5.3)	0
Adrenal disorder (*N* = 8)	4 (50.0)^b^	4 (50.0)	0	0
Neurological disorder (*N* = 6)	2 (33.3)	4 (66.7)	0	0
Severe skin disorder (*N* = 5)	5 (100)	0	0	0
Cardiac disorder (*N* = 5)	2 (40.0)	1 (20.0)	2 (40.0)	0
Renal disorder (*N* = 4)	3 (75.0)	1 (25.0)	0	0
Myasthenia gravis, myocarditis, myositis, rhabdomyolysis (*N* = 2)	2 (100)	0	0	0
Type 1 diabetes mellitus (*N* = 1)	0	1 (100)	0	0

The number (percentage) of patients is shown

*HRT* hormonal replacement therapy

^a^Two patients lacked the treatment data

^b^Patients continuing HRT were included

^c^Two patients recovered with a sequela

^d^One patient lacked the treatment data

### Effectiveness

In the effectiveness analysis set, at least 334 out of 598 patients (55.9%; 95% CI 51.8–59.9%) survived for ≥ 6 months. Specifically, 312 (66.0%), 14 (16.3%), and 8 (20.5%) patients with ECOG PS 0–1, 2, and 3–4 survived for ≥ 6 months, respectively.

## Discussion

This post-marketing surveillance study assessed the safety and effectiveness of nivolumab treatment among patients with head and neck cancer in the real-world setting in Japan.

All patients with head and neck cancer treated with nivolumab were registered in this study without any specific exclusion criteria; thus, this study population likely represents actual patients suffering from head and neck cancer to be treated with nivolumab in Japan. This study included considerably numerous patients who were ≥ 75 years and had ECOG PS ≥ 2; however, no Japanese patients having these categories were included in CheckMate 141 [5]. Likewise, we included patients with tumors in the maxillary sinus or larynx, but no corresponding Japanese patients were included in CheckMate 141. Thus, the safety profile and effectiveness of nivolumab in this study can fill the data gap between the real-world condition and CheckMate 141.

Although patients with ECOG PS ≥ 2 were included, the incidence of grade 3 or 4 TRAEs (9.9%) was similar to that reported in the global (13.1%) and Asian (8.7%) populations in CheckMate 141, and the overall incidence of any TRAEs in our study (36.1%) was even lower than that (58.9%) in CheckMate 141 [4, 5]. Considering the differences between observational studies and clinical trials, the incidences of TRAEs may be underestimated in our study, indicating a potential study limitation. Furthermore, the overall incidence of TRAEs in our study is similar to that of immune-related adverse events reported in Japanese real-world studies [7–9]. Considering that the incidences of each TRAE in our study are mostly comparable to those in CheckMate 141, no additional clinical concern emerged for nivolumab treatment for head and neck cancer. The incidence of TRAEs was higher in patients with any medical histories, possibly attributed to the higher incidence of TRAEs in patients with hepatic, renal, or thyroidal medical history. However, although patients with hepatic and thyroidal medical history had a slightly higher incidence of hepatobiliary disorders and endocrine disorders, respectively, patients with hepatic, renal, or thyroidal medical history demonstrated no marked characteristic of TRAE.

Meanwhile, the incidence of grade 3 or 4 TRAE categories of special interest was apparently low, and most patients with TRAE recovered or remitted; therefore, these TRAEs are manageable with appropriate treatments in the real-world setting. However, some TRAE categories of special interest, such as thyroid dysfunction, hepatic dysfunction, and ILD, were observed not only after the first nivolumab dose but throughout the treatment period. Therefore, these TRAEs should be carefully monitored, while the patient is still treated with nivolumab.

The incidence of ILD (4.1%) in our study population was slightly higher than that reported in the intention-to-treat (ITT) population of CheckMate 141 (3.0%), while the incidences of grade ≥ 3 ILD were similar between these two study populations (1.5% and 1.3%, respectively). The incidence of lung injury induced by anticancer drugs was markedly higher in Japan than worldwide [10]; this finding may be recaptured in the present study. None of the 18 Japanese patients out of 236 global patients in CheckMate 141 reported ILD [5, 11], probably because the number of patients was too small to detect the event. Pneumonitis or pulmonary fibrosis history has been suggested as a risk factor for drug-induced ILD [10]. Consistently, medical histories of pulmonary infection (HR 5.64) and emphysema or chronic obstructive pulmonary diseases (HR 5.11) were the identified risk factors for ILD in our study.

In addition, our study population had higher incidences of thyroid dysfunction (10.2%) and hepatic dysfunction (5.3%) than the ITT population of CheckMate 141 (7.2% and 2.1%, respectively). Patients with head and neck cancer who had undergone radiotherapy for neck cancer or cervical lymph node metastasis often developed thyroid dysfunction, which could be a relatively late-appeared toxicity with median onset of ≥ 2 years after radiotherapy [12, 13]. While approximately 90% of patients in both our study and CheckMate 141 received prior radiotherapy for head and neck cancer, our study had a relatively higher proportion of patients receiving nivolumab as a third-line or later therapy than CheckMate 141 (78.3% vs. 55.8%) [5]. Thus, the average time from prior radiotherapy to nivolumab administration may be longer in this study; this length of time may be associated with the higher incidence of thyroid dysfunction. Meanwhile, although smoking history was a high risk factor for hepatic dysfunction, the possible reason for the higher incidence of hepatic dysfunction in this study remains unclear.

For the study limitations, we did not conduct a central review of TRAEs in case report forms. Thus, TRAE incidences may be underestimated or overestimated compared with those reported in clinical trials.

In conclusion, nivolumab treatment was overall safe and could be beneficial in patients with head and neck cancer in the real-world setting in Japan.

## Supplementary Information

Below is the link to the electronic supplementary material.

Supplementary file1 (DOCX 47 KB)
