# Supplementary material for: Real-world safety and effectiveness of nivolumab for recurrent or metastatic head and neck cancer in Japan: a post-marketing surveillance
Source: Int J Clin Oncol. 2021 Jun 10;26(9):1619–27. doi: 10.1007/s10147-021-01949-1 (PMC8364900; doi:10.1007/s10147-021-01949-1)
Supplement: Supplementary file 1 — Supplementary file1 (DOCX 47 KB) [file 10147_2021_1949_MOESM1_ESM.docx]

**Real-world safety and effectiveness of nivolumab for recurrent or metastatic head and neck cancer in Japan: a post-marketing surveillance**

Makoto Tahara, MD, PhD^1,*^, Naomi Kiyota, MD, PhD^2,3^, Ken-ichi Nibu, MD, PhD^4^, Ayumi Akamatsu^5^, Tomohiro Hoshino^6^, and Ryuichi Hayashi, MD^7^.

^*^Correspondence to: Makoto Tahara, MD, PhD

Department of Head and Neck Medical Oncology, National Cancer Center Hospital East, 6-5-1 Kashiwanoha, Kashiwa, Chiba, 277-8577, Japan

Tel +81-4-7133-1111

Fax +81-4-7131-9960

Email matahara@east.ncc.go.jp

Supplementary Table S1 Treatment-related adverse events occurring in 1% or more patients

| **Treatment-related adverse events^a^** | **This study**  **(N = 607)** | **CheckMate 141**  **(Ferris et al, 2016)**  **(N = 236)** |
| --- | --- | --- |
| **Any** | **219 (36.1)** | **139 (58.9)** |
| **Endocrine disorder** | **62 (10.2)** | **14 (5.9)** |
| Hypothyroidism | 46 (7.6) | 9 (3.8) |
| **General disorders and administration site conditions** | **39 (6.4)** | **57 (24.2)** |
| Pyrexia | 17 (2.8) | 4 (1.7) |
| Malaise | 14 (2.3) | 2 (0.8) |
| Fatigue | 4 (0.7) | 33 (14.0) |
| Edema peripheral | 2 (0.3) | 3 (1.3) |
| Asthenia | 0 | 10 (4.2) |
| Influenza like illness | 0 | 3 (1.3) |
| Mucosal inflammation | 0 | 3 (1.3) |
| **Respiratory, thoracic and mediastinal disorders** | **37 (6.1)** | **19 (8.1)** |
| Interstitial lung disease | 20 (3.3) | 0 |
| Pneumonitis | 1 (0.2) | 5 (2.1) |
| Cough | 0 | 6 (2.5) |
| **Skin and subcutaneous tissue disorders** | **37 (6.1)** | **43 (18.2)** |
| Rash | 15 (2.5) | 18 (7.6) |
| Pruritus | 3 (0.5) | 17 (7.2) |
| Dry skin | 2 (0.3) | 7 (3.0) |
| Rash maculo-papular | 2 (0.3) | 5 (2.1) |
| Skin lesion | 0 | 3 (1.3) |
| **Investigations** | **36 (5.9)** | **30 (12.7)** |
| Aspartate aminotransferase increased | 16 (2.6) | 2 (0.8) |
| Alanine aminotransferase increased | 10 (1.6) | 2 (0.8) |
| Blood thyroid stimulating hormone increased | 4 (0.7) | 3 (1.3) |
| Platelet count decreased | 3 (0.5) | 4 (1.7) |
| Amylase increased | 0 | 8 (3.4) |
| Lipase increased | 0 | 6 (2.5) |
| Weight decreased | 0 | 4 (1.7) |
| **Gastrointestinal disorders** | **34 (5.6)** | **47 (19.9)** |
| Diarrhea | 18 (3.0) | 16 (6.8) |
| Nausea | 5 (0.8) | 20 (8.5) |
| Vomiting | 2 (0.3) | 8 (3.4) |
| Stomatitis | 2 (0.3) | 5 (2.1) |
| Dysphagia | 1 (0.2) | 4 (1.7) |
| Constipation | 1 (0.2) | 3 (1.3) |
| **Metabolism and nutrition disorders** | **19 (3.1)** | **32 (13.6)** |
| Decreased appetite | 12 (2.0) | 17 (7.2) |
| Hyponatremia | 1 (0.2) | 4 (1.7) |
| **Hepatobiliary disorders** | **16 (2.6)** | **0** |
| Hepatic function abnormal | 10 (1.6) | 0 |
| **Nervous system disorders** | **13 (2.1)** | **7 (3.0)** |
| Headache | 1 (0.2) | 4 (1.7) |
| **Musculoskeletal and connective tissue disorders** | **11 (1.8)** | **9 (3.8)** |
| Arthritis | 3 (0.5) | 5 (2.1) |

Supplementary Table S1. (continued)

| **Treatment-related adverse events^a^** | **This study**  **(N = 607)** | **CheckMate 141**  **(Ferris et al, 2016)**  **(N = 236)** |
| --- | --- | --- |
| **Infections and infestations** | **9 (1.5)** | **8 (3.4)** |
| **Injury, poisoning and procedural complications** | **8 (1.3)** | **4 (1.7)** |
| Infusion related reaction | 7 (1.2) | 3 (1.3) |
| **Renal and urinary disorders** | **6 (1.0)** | **1 (0.4)** |
| **Blood and lymphatic system disorders** | **5 (0.8)** | **17 (7.2)** |
| Anemia | 3 (0.5) | 12 (5.1) |
| Lymphopenia | 0 | 5 (2.1) |
| **Cardiac disorders** | **5 (0.8)** | **0** |
| **Neoplasms benign, malignant and unspecified** | **4 (0.7)** | **3 (1.3)** |
| **Psychiatric disorders** | **2 (0.3)** | **2 (0.9)** |
| **Eye disorders** | **2 (0.3)** | **2 (0.9)** |
| **Ear and labyrinth disorders** | **1 (0.2)** | **1 (0.4)** |
| **Reproductive system and breast disorders** | **1 (0.2)** | **0** |
| **Vascular disorders** | **0** | **5 (2.1)** |
| **Immune system disorders** | **0** | **1 (0.4)** |

The number (percentage) of patients is shown.

^a^All system organ classes (bold) that are observed in any patients are shown. Only the selected preferred terms that were observed in ≥1% patients in either this study or CheckMate 141 are shown.

Supplementary Table S2 Treatment-related adverse events in patient subpopulations (N = 607)

| **Stratification factors** | **All patients** | **Patients experiencing any TRAEs** | | |
| --- | --- | --- | --- | --- |
|  | **n (%)** | **n (%)** | **95% CI^a^** | **P value^b^** |
| **Sex** | | | | |
| Male | 454 (74.8) | 165 (36.3) | 31.9–41.0 | 0.8461 |
| Female | 153 (25.2) | 54 (35.3) | 27.7–43.4 |  |
| **Age** | | | | |
| ≤64 years | 315 (51.9) | 105 (33.3) | 28.1–38.8 | 0.1532 |
| 65–74 years | 216 (35.6) | 84 (38.9) | 32.3–45.7 |  |
| ≥75 years | 76 (12.5) | 30 (39.5) | 28.4–51.4 |  |
| **ECOG PS** | | | | |
| 0–1 | 478 (78.7) | 179 (37.4) | 33.1–42.0 | 0.1770 |
| 2–4 | 129 (21.3) | 40 (31.0) | 23.2–39.7 |  |
| **Smoking** | | | | |
| Smoker | 408 (67.2) | 151 (37.0) | 32.3–41.9 | 0.2381 |
| Non-smoker | 156 (25.7) | 49 (31.4) | 24.2–39.3 |  |
| Unknown | 43 (7.1) | 19 (44.2) | ND |  |
| **Alcohol** | | | | |
| Drinker | 381 (62.8) | 133 (34.9) | 30.1–39.9 | 0.4911 |
| Non-drinker | 159 (26.2) | 61 (38.4) | 30.8–46.4 |  |
| Unknown | 67 (11.0) | 25 (37.3) | ND |  |
| **Medical history** | | | | |
| Yes | 363 (59.8) | 155 (42.7) | 37.6–48.0 | < 0.0001* |
| No | 242 (39.9) | 64 (26.4) | 21.0–32.5 |  |
| Unknown | 2 (0.3) | 0 | ND |  |
| **Medical history of hepatic diseases** | | | | |
| Yes | 50 (8.2) | 25 (50.0) | 35.5–64.5 | 0.0448* |
| No | 556 (91.6) | 194 (34.9) | 30.9–39.0 |  |
| Unknown | 1 (0.2) | 0 | ND |  |
| **Medical history of renal diseases** | | | | |
| Yes | 32 (5.3) | 18 (56.3) | 37.7–73.6 | 0.0217* |
| No | 573 (94.4) | 200 (34.9) | 30.9–39.0 |  |
| Unknown | 2 (0.3) | 1 (50.0) | ND |  |
| **Medical history of lung diseases** | | | | |
| Yes | 105 (17.3) | 43 (41.0) | 31.5–51.0 | 0.2644 |
| No | 500 (82.4) | 175 (35.0) | 30.8–39.4 |  |
| Unknown | 2 (0.3) | 1 (50.0) | ND |  |
| **Medical history of thyroidal diseases** | | | | |
| Yes | 74 (12.2) | 39 (52.7) | 40.7–64.4 | 0.0028* |
| No | 531 (87.5) | 180 (33.9) | 29.9–38.1 |  |
| Unknown | 2 (0.3) | 0 | ND |  |
| **Medical history of autoimmune disease** | | | | |
| Yes | 13 (2.1) | 6 (46.2) | 19.2–74.9 | 0.5609 |
| No | 593 (97.7) | 213 (35.9) | 32.1–39.9 |  |
| Unknown | 1 (0.2) | 0 | ND |  |

Supplementary Table S2 (continued)

|  | **All patients** | **Patients experiencing any TRAEs** | | |
| --- | --- | --- | --- | --- |
|  | **n (%)** | **n (%)** | **95% CI^a^** | **P value^b^** |
| **Metastasis** | | | | |
| Yes | 382 (62.9) | 141 (36.9) | 32.1–42.0 | 0.5265 |
| No | 203 (33.4) | 69 (34.0) | 27.5–41.0 |  |
| Unknown | 22 (3.6) | 9 (40.9) | ND |  |
| **Surgery of HNC** | | | | |
| Yes | 349 (57.5) | 136 (39.0) | 33.8–44.3 | 0.0880 |
| No | 258 (42.5) | 83 (32.2) | 26.5–38.2 |  |
| **Radiotherapy for HNC** | | | | |
| Yes | 558 (91.9) | 199 (35.7) | 31.7–39.8 | 0.4353 |
| No | 48 (7.9) | 20 (41.7) | 27.6–56.8 |  |
| Unknown | 1 (0.2) | 0 | ND |  |
| **Treatment line** | | | | |
| 1 | 2 (0.3) | 2 (100) | 15.8–100 | 0.6796 |
| 2 | 126 (20.8) | 42 (33.3) | 25.2–42.3 |  |
| ≥3 | 475 (78.3) | 174 (36.6) | 32.3–41.1 |  |
| Unknown | 4 (0.7) | 1 (25.0) | ND |  |
| **Time after the last dose of platinum agents to progression or recurrence** | | | | |
| ≤6 months | 375 (62.7) | 136 (36.3) | 31.4–41.4 | 0.9525 |
| >6 months | 211 (35.3) | 76 (36.0) | 29.5–42.9 |  |
| Unknown | 12 (2.0) | 3 (25.0) | ND |  |
| **Number of nivolumab administration** | | | | |
| 1–4 | 224 (36.9) | 75 (33.5) | 27.3–40.1 | 0.3743 |
| 5–8 | 159 (26.2) | 58 (36.5) | 29.0–44.5 |  |
| 9–12 | 78 (12.9) | 33 (42.3) | 31.2–54.0 |  |
| ≥13 | 146 (24.1) | 53 (36.3) | 28.5–44.7 |  |

^a^95% CIs were calculated by Fisher’s exact test.

^b^P values were calculated by Fisher’s exact test (sex, smoking, alcohol, medical history, medical history of hepatic, renal, lung, thyroidal, or autoimmune disease, metastasis, surgery of HNC, and Radiotherapy for HNC) or the Wilcoxon rank sum test (age, ECOG PS, treatment line, time after the last dose of platinum agents to progression or recurrence, and number of nivolumab administration).

*, statistically significant; CI, confidence interval; ECOG PS, Eastern Cooperative Oncology Group performance status; HNC, head and neck cancer; ND, not determined; TRAE, treatment-related adverse event.

| **Treatment-related adverse events** | **Medical history** | | | | | | | |
| --- | --- | --- | --- | --- | --- | --- | --- | --- |
|  | **Any** | | **Liver** | | **Kidney** | | **Thyroid** | |
|  | **No**  **n = 242** | **Yes**  **n = 363** | **No**  **n = 556** | **Yes**  **n = 50** | **No**  **n = 573** | **Yes**  **n = 32** | **No**  **n = 531** | **Yes**  **n = 74** |
| Any | 64 (26.4) | 155 (42.7) | 194 (34.9) | 25 (50.0) | 200 (34.9) | 18 (56.3) | 180 (33.9) | 39 (52.7) |
| Endocrine disorder | 20 (8.3) | 42 (11.6) | 56 (10.1) | 6 (12.0) | 58 (10.1) | 4 (12.5) | 47 (8.9) | 15 (20.3) |
| Respiratory, thoracic and mediastinal disorders | 8 (3.3) | 29 (8.0) | 31 (5.6) | 6 (12.0) | 34 (5.9) | 2 (6.3) | 30 (5.6) | 7 (9.5) |
| General disorders and administration site conditions | 7 (2.9) | 32 (8.8) | 33 (5.9) | 6 (12.0) | 35 (6.1) | 4 (12.5) | 32 (6.0) | 7 (9.5) |
| Gastrointestinal disorders | 12 (5.0) | 22 (6.1) | 32 (5.8) | 2 (4.0) | 32 (5.6) | 2 (6.3) | 30 (5.6) | 4 (5.4) |
| Investigations | 9 (3.7) | 27 (7.4) | 35 (6.3) | 1 (2.0) | 32 (5.6) | 4 (12.5) | 35 (6.6) | 1 (1.4) |
| Skin and subcutaneous tissue disorders | 10 (4.1) | 27 (7.4) | 33 (5.9) | 4 (8.0) | 32 (5.6) | 5 (15.6) | 29 (5.5) | 8 (10.8) |
| Metabolism and nutrition disorders | 2 (0.8) | 17 (4.7) | 17 (3.1) | 2 (4.0) | 16 (2.8) | 3 (9.4) | 14 (2.6) | 5 (6.8) |
| Hepatobiliary disorders | 2 (0.8) | 14 (3.9) | 13 (2.3) | 3 (6.0) | 14 (2.4) | 2 (6.3) | 13 (2.4) | 3 (4.1) |
| Injury, poisoning and procedural complications | 0 | 8 (2.2) | 7 (1.3) | 1 (2.0) | 7 (1.2) | 1 (3.1) | 5 (0.9) | 3 (4.1) |
| Musculoskeletal and connective tissue disorders | 2 (0.8) | 9 (2.5) | 10 (1.8) | 1 (2.0) | 10 (1.7) | 1 (3.1) | 8 (1.5) | 3 (4.1) |
| Blood and lymphatic system disorders | 0 | 5 (1.4) | 5 (0.9) | 0 | 5 (0.9) | 0 | 5 (0.9) | 0 |
| Renal and urinary disorders | 3 (1.2) | 3 (0.8) | 5 (0.9) | 1 (2.0) | 4 (0.7) | 2 (6.3) | 4 (0.8) | 2 (2.7) |
| Infections and infestations | 3 (1.2) | 6 (1.7) | 7 (1.3) | 2 (4.0) | 8 (1.4) | 1 (3.1) | 8 (1.5) | 1 (1.4) |
| Cardiac disorders | 2 (0.8) | 3 (0.8) | 4 (0.7) | 1 (2.0) | 3 (0.5) | 2 (6.3) | 5 (0.9) | 0 |
| Neoplasms benign, malignant and unspecified | 1 (0.4) | 3 (0.8) | 3 (0.5) | 1 (2.0) | 4 (0.7) | 0 | 2 (0.4) | 2 (2.7) |
| Nervous system disorders | 3 (1.2) | 10 (2.8) | 11 (2.0) | 2 (4.0) | 12 (2.1) | 1 (3.1) | 12 (2.3) | 1 (1.4) |
| Eye disorders | 1 (0.4) | 1 (0.3) | 1 (0.2) | 1 (2.0) | 2 (0.3) | 0 | 2 (0.4) | 0 |
| Psychiatric disorders | 1 (0.4) | 1 (0.3) | 2 (0.4) | 0 | 2 (0.3) | 0 | 2 (0.4) | 0 |
| Ear and labyrinth disorders | 0 | 1 (0.3) | 1 (0.2) | 0 | 1 (0.2) | 0 | 1 (0.2) | 0 |
| Reproductive system and breast disorders | 0 | 1 (0.3) | 1 (0.2) | 0 | 1 (0.2) | 0 | 0 | 1 (1.4) |

Supplementary Table S3 Treatment-related adverse events in patients with medical history

The number (percentage) of patients is shown.

Supplementary Table S4 Treatment-related adverse events of special interest

| **Categories** | **This study**  **(N = 607)** | | **CheckMate 141**  **ITT population^a^**  **(Ferris et al, 2016)**  **(N = 236)** | | **CheckMate 141**  **Japanese population^a^**  **(Kiyota et al, 2017)**  **(N = 18)** | |
| --- | --- | --- | --- | --- | --- | --- |
|  | **Any grades** | **Grade 3**  **or greater** | **Any grades** | **Grade 3**  **or greater** | **Any grades** | **Grade 3**  **or greater** |
| Thyroid dysfunction | 62 (10.2) | 4 (0.7) | 17 (7.2) | 1 (0.4) | 2 (11.1) | 0 |
| Hepatic dysfunction | 32 (5.3) | 11 (1.8) | 5 (2.1) | 2 (0.8) | 0 | 0 |
| Interstitial lung disease | 25 (4.1) | 9 (1.5) | 7 (3.0) | 3 (1.3) | 0 | 0 |
| Colitis, severe diarrhea | 21 (3.5) | 7 (1.2) | 16 (6.8) | 0 | 1 (5.6) | 0 |
| Infusion reaction | 19 (3.1) | 1 (0.2) | NA | NA | NA | NA |
| Adrenal disorder | 8 (1.3) | 2 (0.3) | 1 (0.4) | 1 (0.4) | 0 | 0 |
| Neurological disorder | 6 (1.0) | 0 | 2 (0.8) | 0 | 0 | 0 |
| Severe skin disorder | 5 (0.8) | 5 (0.8) | 1 (0.4) | 0 | 0 | 0 |
| Cardiac disorder | 5 (0.8) | 2 (0.3) | 0 | 0 | 0 | 0 |
| Renal disorder | 4 (0.7) | 1 (0.2) | 1 (0.4) | 0 | 0 | 0 |
| Myasthenia gravis, myocarditis, myositis, rhabdomyolysis | 2 (0.3) | 1 (0.2) | 0 | 0 | 0 | 0 |
| Type 1 diabetes mellitus | 1 (0.2) | 1 (0.2) | 0 | 0 | 0 | 0 |
| Venous thromboembolism | 0 | 0 | 1 (0.4) | 1 (0.4) | 0 | 0 |

The number (percentage) of patients is shown.

^a^Patients in CheckMate 141 who experienced each treatment-related adverse events between the first dose and 30 days after the lase dose were counted.

NA, not available.

Supplementary Table S5 Risk factors evaluated by multivariate analysis

| **Factors** | **Comparison** | **Hazard ratio (95% CI)** |
| --- | --- | --- |
| **Factors for thyroid dysfunction^a^** | | |
| Gender | Male vs Female | 0.62 (0.32–1.22) |
| Age group | ≥75 years vs  ≤74 years | 0.55 (0.17–1.79) |
| ECOG PS | 2–4 vs 0–1 | 0.33 (0.12– 0.91) |
| Smoking history | Current or former vs never | 2.38 (1.08–5.25) |
| Medical history of thyroidal diseases | Yes vs No | 1.81 (0.89–3.66) |
| Medical history of autoimmune diseases | Yes vs No | 2.50 (0.63–10.01) |
| Metastasis | Yes vs No | 1.37 (0.72–2.60) |
| Treatment line | 2nd vs ≥3rd | 1.17 (0.60–2.29) |
| **Factors for hepatic dysfunction^a^** | | |
| Gender | Male vs Female | 0.98 (0.39–2.47) |
| Age group | ≥75 years vs  ≤74 years | 0.89 (0.28–2.88) |
| ECOG PS | 2–4 vs 0–1 | 1.86 (0.85–4.06) |
| Smoking history | Current or former vs never | 3.10 (1.09–8.82) |
| Medical history of hepatic diseases | Yes vs No | 1.21 (0.36–4.03) |
| Medical history of autoimmune diseases | Yes vs No | 2.22 (0.26–18.85) |
| Metastasis | Yes vs No | 1.64 (0.71–3.78) |
| Treatment line | 2nd vs ≥3rd | 1.57 (0.72–3.42) |
| **Factors for ILD^b,c^** | | |
| Medical history of emphysema or COPD | Yes vs No | 5.11 (1.30–20.05) |
| Medical history of pulmonary infection | Yes vs No | 5.64 (1.74–18.35) |

For all risk factor analyses, factors with a missing value of ≥10% and variables with Spearman's rank correlation coefficient of ≥0.95 were not included.

^a^Among the factors extracted by univariate analysis, clinically relevant factors listed in this table were used as explanatory variables in multivariate analysis.

^b^Univariate analysis for ILD extracted the medical histories of emphysema or COPD and pulmonary infection, with a hazard ratio of >2; these two factors are considered as risk factors, which were used as explanatory variables in multivariate analysis.

^c^Each of clinically relevant factors extracted from univariate analysis (sex, age group, ECOG PS, smoking history, medical history of ILD, medical history of autoimmune disease, metastasis, radiotherapy for HNC, experience of molecular targeted drugs, treatment line) was individually analyzed with the two identified risk factors for ILD (a medical history of emphysema or COPD and a medical history of pulmonary infection) as explanatory variables in multivariate analyses. However, no further significant risk factors were found.

CI, confidence interval; COPD, chronic obstructive pulmonary disease: ECOG PS, Eastern Cooperative Oncology Group performance status; HNC, head and neck cancer; ILD, interstitial lung disease
